# Formulation, *In Vitro* and *In Vivo* Pharmacokinetics of Anti-HIV Vaginal Bioadhesive Gel

**DOI:** 10.4103/0975-1483.80290

**Published:** 2011

**Authors:** A Chatterjee, B B Bhowmik, Y S Thakur

**Affiliations:** *Department of Pharmaceutics, Himalayan Pharmacy Institute, Majhitar, Rangpo, East Sikkim, India*

**Keywords:** Bioadhesive, human immunodeficiency virus, pharmacokinetics, vaginal gel, zidovudine

## Abstract

Inexpensive and female-controlled pre-exposure prophylaxis strategies to prevent mucosal transmission of the virus, is urgently needed with the rising prevalence of human immunodeficiency virus (HIV-1 and HIV2) infections in women. Zidovudine-loaded bioadhesive vaginal gel may become one of the very useful strategies, as it can be used not only for controlled release but also for enhancing bioavailability. Drug delivery through vaginal gel is a promising area for continued research with the aim of achieving controlled release with enhanced bioavailability over longer periods of time. The aim of the study was to develop a newer prolong releasing Zidovudine (AZT) bioadhesive vaginal gel to treat HIV infections with increased patient convenience. AZT-loaded bioadhesive vaginal gel was prepared successfully by using cold mechanical method. F3 formulation containing carbopol–HPMC (1:3) was selected and evaluated in order to achieve objectives of this study. *In vitro* drug release study of F3 showed in 24 h drug released following case I Fickian (*n* ≤ 0.5) transport mechanism, and *in vivo* drug release was found much better (T_max_), (C_max_), and bioavailability (*F*) comparison with oral pour drug solution. It was also showed good extrudability, spreadability, and bioadhesive strength. A generalized protocol, for the further research, in this area will surely expected to yield significant outcome with improved drug delivery system.

## INTRODUCTION

The women (15.4 million) are approximately 50% of people (33.2 million) infected and living with HIV, as reported in 2007 UN AIDS summary.[1] In most regions of the world, HIV is affecting women and girls in increasing numbers. Vaginal drug delivery is a very challenging and less explored research area. Gel as dosage forms were successfully used as drug delivery systems with their ability to prolong the drug release. Topical, self-administered products containing HIV microbicides were aimed to prevent and to reduce HIV infection in women and may represent the most promising strategy for combating the HIV/AIDS epidemic at the present time. The vagina is an efficient route[2] for drug administration due to presence of dense blood vessels network and avoids first-pass.[3,4] Ideally, anti-HIV vaginal gels (F) should adheres in vaginal medium; provide uniform drug–hydrogel coating of vaginal tissue, resulting in intravaginal biomimetic lubrication during intercourse, and retention of this gel layer before and after intercourse. Most importantly, controlled release of anti-HIV drugs form this gel inactivates the viral load potentially introduced during sexual activity. Zidovudine (AZT) with short elimination half-life of about 1 h, high dose (250 mg in every 4 h while 300 mg twice a day, in some cases), low systemic bioavailability (64%) due to rapid hepatic fast-pass metabolism, was chosen as a model drug of choice.[5] The use of prolong-release bioadhesive vaginal gel was thought to offer numerous benefits including prolong residence time of the dosage form at the site of absorption due to bioadhesion to the vaginal mucosa, prolong drug release, improved bioavailability and decreased side effect of drug, and ultimately improved patient compliance. Keeping in view of the above uniqueness, this study was designed to develop a newer formulation for prolong release of AZT to treat HIV infections with increased patient convenience.

## MATERIALS AND METHODS

### Materials

Zidovudine was obtained from Arbindo Pharmaceutical Ltd. (Hydrabad, India). Carbopol 940P were received as gift sample from Corel Pharma Chem (Ahmedabad, India). HPMCK4M was obtained from LOBA Chemicals (Kolkata, India). All other chemicals and reagents used were of analytical grade and used as received.

### Methods

#### Preparation of Zidovudine-loaded vaginal gel

Vaginal gels were prepared by cold mechanical method described by Schmolka.[6,7] The required quantity of drug (AZT) and polymer (Carbopol 940P and HPMCK4M) was weighed, then it was sprinkled slowly on surface of purified water for 2 h. After that it was continuously stirred by mechanical stirrer, till the polymer was soaked in the water. Finally solution was kept for overnight for complete hydration of polymer. With continuous stirring, triethanolamine was added to neutralize the gel and to maintain the pH of the gel. Now the appropriate quantity of dimethyl sulphoxide (DMSO) was added to the gel, which behaved as the penetration enhancer, followed by addition of required quantity of ethanol to make the soft gel. Care should be taken to avoid incorporation of air into gel. In this way, four formulations (F1–F4) of gel were prepared by combinations of carbopol 940 and HPMC. Finally, the preparations were packed in wide mouth plastic jar covered with screw capped plastic lid after covering the mouth with an aluminum foil and were kept in dark and cool place. The formulations were preserved for further study. The generalized bioadhesive gel preparation protocol depends on choice of solvent, stirring speed of mechanical stirrer, and optimization at every preparative steps. AZT incorporated into bioadhesive gel intended for vaginal was successfully prepared using mechanical stirring technique as designed in Table 1.

**Table 1:** Formulation design of bioadhesive vaginal gels (F1–F4)

Ingredients	Formulation			
	F1	F2	F3	F4
Zidouvdine (mg)	100	100	100	100
Carbopol (mg)	100	100	100	100
HPMC (mg)	100	200	300	400
DMSO (mL)	0.2	0.2	0.2	0.2
Triethanolamine (mL)	0.9	0.9	0.9	0.9
Methyl Paraben (mg)	15	15	15	15
Alcohol (mL)	5.0	5.0	5.0	5.0
Water (up to mL)	100	100	100	100

#### Percent yield of vaginal gel

The percent yield was calculated[8] as the weight of the formulations (Fs) recovered from each batch divided by total weight of drug containing microparticles and other all ingredients used to prepare Fs multiplied by 100. The percentage yield of each formulation was replicated three times. The yield of Fs was calculated using the following formula:

$$Y\;=\;\left\{P_{m}\;-\;Z_{g}\right\}/T_{m}\left[P\;+\;Ig\right]\times 100,$$

where *Y* = yield, *P*_m_ = practical mass, *Z*_G_ = vaginal gel, *T*_m_ = theoretical mass, *P* = polymer, and Ig = ingredients.

#### Drug content evaluation

Drug content was determined by Sanjay *et al*.,[9] dissolved accurately weighed quantity of bioadhesive vaginal gel with 20 mL of simulated vaginal fluid (SVF, acetate buffer I.P. pH 4.7) in a 50 mL of volumetric flask with continuous stirring. The volume was adjusted up to 50 mL with SVF. Blank bases were also treated in similar manner for blank determination. Both the test sample and blank were allowed to stand on mechanical stirrer for 2 h and kept for 24 h. In the next day, all the solutions were filtered using Whatmann filter paper no. 44. Then, the filtrate solution was kept as a stock solution. After suitable dilution, the absorbance of the solution with the blank was measured by UV-visible spectrophotometer (UV-1700, Shimadzu, Japan) at 267 nm. Drug content was calculated from the standard calibration curve, *Y* = 0.038 × *X*, where *Y* = absorbance and *X* = concentration of the drug. *D*_c_ = [*C*_c_ ×D_f_ × *V*]/*C*_F_, where as *D*_c_= drug content, *C*_C_= concentration, *D*_F_= dilution factor, *V* = volume taken, and *C*_F_= conversion factor.

#### Bioadhesive strength of Fs using isolated goat vagina

Isolated goat vaginal tissue (*Capra hircus*, local breed, obtained immediately after killing of animals at a slaughter house) was cleaned and then separated from the supporting muscular and connective tissues taking care to maintain integrity of mucosa, and kept at 0°C till further use. Before experiments, goat vaginal tissue was thawed in normal saline. The bioadhesion measurement was performed using a modified balance method intact with freshly excised goat vaginal mucosal membrane as an *in vitro* model.[10] The two pans of physical balance were removed. Right side pan was replaced with a 100 mL beaker and on left side, a glass slide was hanged. For balancing the assembly, a weight of 20 g was hanged on left side. Another glass slide was placed below the hanged slide. Portions of vaginal membranes were attached with both slides. The height of this setup was so adjusted, in such a way that it leaves a space of about 0.2 cm between two vaginal membrane faces. One gram of gel was placed between two vaginal membrane faces. Little pressure was applied to form bioadhesion bond, and then slowly drop of water was added on right side beaker, till the gel was separated from one face of vaginal membranes attached. Volume of water added was converted to mass. This gave the bioadhesive strength of gel in gm. An initial investigation examined the reproducibility of the system using five same formulations. Then, the study was carried out for all formulations.[11]

#### Spreadability of vaginal gels

Spreadability was determined[12] by apparatus suggested by Mutimer which was suitably modified in the laboratory and used for the study. It consists of a wooden block, which was provided by a pulley at one end. By this method, spreadability was measured on the basis of “Slip” and “Drag” characteristics of gels. A ground glass slide was fixed on this block. An excess of gel about 2 g under study was placed on this ground slide. The gel was then sandwiched between this slide and another glass slide having the dimension of fixed ground slide and provided with the hook. One kilogram weight was placed on the top of the two slides for 5 min to expel the air and to provide a uniform film of the gel between the slides. Excess of the gel was scrapped off from the edges. The top plate was then subjected to pull of 80 g. With the help of string attached to the hook and the time (in seconds) required by the top slide to cover a distance of 7.5 cm be noted. A shorter interval indicates better spreadability.[13] Spreadability was then calculated using the following formula:

$$S\;=\;M_{g}\;\times \;L_{s}/T_{m},$$

where *S* is the spreadability, *M*_g_ is the weight in the pan (tied to the upper slide), *L*_s_ is the length moved by the glass slide, and T_m_ represents the time taken to separate the slide completely from each other.

#### Extrudability of vaginal gels

It is a usual empirical test to measure the force required to extrude the material from tube. The method applied for determination[13] of applied shear in the region of the rheogram corresponding to a shear rate exceeding the yield value and exhibiting consequent plug flow one such apparatus is described by Chakole.In this study, the method adopted for evaluating gel formulation for extrudability was based upon the quantity in percentage of gel and gel extruded from lacquered aluminum collapsible tube on application of weight in grams required to extrude at least 0.5 cm ribbon of gel in 10 s. The measurement of extrudability of each formulation was in triplicate and the average values are presented. The extrudability was than calculated using the following formula:

$$E_{p}\;=\;G_{m}/A,$$

where *E*_P_ is extrudability, *G*_m_ is applied weight to extrude gel from tube (in g) and *A* is area (in cm ^2^).

#### In vitro drug diffusion studies of vaginal gel

*In vitro* drug diffusion studies were carried out using biochambered donor receiver compartment model, Keshery–Chien diffusion cell. Cellophane membrane was stored in SVF. The cellophane membrane acts like a barrier between the gel and the SVF (sink phase). One gram of gel was placed on the surface of processed cellophane membrane, and membrane was fixed to one end of the cylindrical donor compartment by adhesive tape such that the lower end of tube containing film just touched the surface of SVF medium. In addition, 0.5 mL of SVF was placed and maintained at same level throughout the study in the donor compartment. Temperature was maintained at 37 ± 2°C with constant stirring at 50 ± 10 rpm. A quantity of 5 mL sample was withdrawn from the receptor compartment at definite time interval and replaced with 5 mL of SVF to maintain constant volume. The drug was estimated by using Schimadzu UV–Visible spectrophotometer at 267 nm (λ_max_).[14]

#### Drug release kinetic studies of gel formulations

To study the exact mechanism of drug release from the vaginal bioadhesive gel, drug release data were analyzed according to zero-order, first-order, Higuchi square root, and Korsmeyer–Peppa’s equations. The criterion for selecting the most appropriate model was chosen on the basis of goodness of fit test.[15,16] To investigate the mechanism of AZT release from gel formulations, the release data were analyzed with the following mathematical models: Zero-order kinetic (equation a), first-order kinetic (equation b), and Higuchi kinetic (equation c).

(a) $$Q_{t}\;=\;K_{0}t$$

(b) $$\ln\;Q_{t}\;=\;\ln\;Q_{0}\;-\;K_{1}t$$

(c) $$Q_{t}\;=\;K_{h}t^{1/2}$$

The following plots were made: *Q*_t_ versus *t* (zero-order kinetic model), ln (*Q*_0_ – *Q*_t_) versus *t* (first-order kinetic model), and Q_t_ versus *t*^1/2^. Here, *Q*_t_ is the percent of drug released at time *t*, *Q*_0_ is the percent of drug present in the microparticles, *K*_0_, *K*_1_, and *K*_h_ are the constants of the equations.

Further, to confirm the mechanism of drug release, the first 60% of drug release was fitted in Korsmeyer-Peppas model (equation d):

$$M_{t}/M_{\;}\;=\;K_{p}t^{n},$$

where (*M* _t_ / *M*_α_) is the fraction of the drug release at time (*t*), *K*_P_ is the rate constant, *n* the value is used to characterize different release mechanisms, and is calculated from the slope of log of fraction of drug released (*M* _t_ /*M*_α_) versus of time (*t*).

The following plots were made: cumulative % drug release versus time (zero-order kinetic models); log cumulative of % drug remaining versus time (first-order kinetic model); cumulative % drug release versus square root of time (Higuchi model); and log cumulative % of drug released versus log time (Korsmeyer–Peppas model).

### *In vivo* study of vaginal gel

#### Experimental design

Eight adult female rabbits (New Zealand white species) weighing 1.5–1.7 kg were used for this study. The animals were divided into two groups both containing four animals each, and one animal was used as control. The animals were kept fasted for overnight. Water was given *ad libitum* during fasting and throughout experiment. The rabbits were not anesthetized during or prior to the experiment and were applied the formulation with the help of vaginal applicator and standard oral dose of pour drug with the help of oral cannula. The procedures employed in this study were approved by Institutional Ethical Committee (no: HPI/07/60/IAEC/0013). One group was fed with standard AZT at a dose of 2 mg. Two grams of vaginal gel (F3) was applied to other group with the help of vaginal applicator and marked as test “Formulationx”. Blood samples (2 mL) were collected from marginal ear vein at an interval of 1, 2, 4, 6, and 24 h during this study. The same method was followed for each group (both standard and test). The blood samples withdrawn as above were transferred to a series of graduated centrifuge tube containing 1 mL of 10% w/v EDTA solution.[17] The samples were centrifuged immediately at 3000rpm for 15 min in cooling centrifuge machine to collect plasma.[18] The plasma was separated and transferred into other set of sample tubes and stored at –20°C until assayed. About 1 mL plasma was mixed with 1 mL of 15 w/v% trichloro acidic acid (TCA), shaken well for 3 min and centrifuged at 3000rpm for 15 min. The plasma samples were analyzed for AZT by first passing the plasma samples through Silica gel column and analyzed by HPLC (LC-20 AT, Shimadzu, Japan) using mobile phase methanol:water (60:40)[19] at a flow rate of 1.2 mL/min. Twenty microliters of injection volume was eluted in RP C(18) column (4.6 mm × 150 mm) at room temperature. The column eluted was monitored at wavelength (λ_max_) 267 nm using diode array UV detector.

#### Pharmacokinetics analysis

The highest observed concentration during the study period; *C*_max_ and time, at which *C*_max_ observed, *T*_max_, were obtained directly from the plasma concentration time profiles. The area under the plasma concentration time curve (AUC _0-24h_, and AUC _0-α_, μg h/mL) was calculated based on the trapezoidal rule. The volume of distribution (*V*_d_), total body clearance (Cl _T_), elimination rate constant (*K*_E_), and half-life (*t*_1/2_) was also calculated.[20]

#### Statistical analysis

Analysis of Variance is presented as (mean+SD) and (*P*=0.05) was considered significant. Statistical data analyses were performed by statistical analysis using MYSTAT soft-ware the ANOVA one way at 5% level of significance *P* < 0.05.

## RESULTS AND DISCUSSION

The generalized protocol depends on choice of ingredient, successful preparation of vaginal gels, and optimization at every preparative steps. The formulation code and composition of vaginal gel were presented in Table 1.

### Percent yield, drug content, spreadability, extrudability, and bioadhesive strength of vaginal gels

The percent yields of Fs were calculated and found to be into the range of 98.97 ± 3.36 to 99.90 ± 0.05%w/w given in column 2 of Table 2.The drug content of Fs was found to be in the range of 96.24 ± 0.09 to 100.01 ± 0.19 mg/100 g of gel as in column 3 of Table 2. Observations for spreadability study are in column 5 of Table 2. Spreadability of all vaginal gels of all Fs was in the range of 119.29 ± 0.02 to 158.12 ± 0.24g cm/s. Extrudability study shown in column 4 of Table 2 was in the range of 84.0 ± 1.78 to 97.0 ± 2.93 g/cm^2^. As the concentration of polymer increased the extrudability of Fs also decreased, because as the concentration of polymer increased weight required to extrude gel from tube also increases. Vaginal bioadhesive strengths of all Fs, using goat vagina, were found in the following order F4 > F3 > F2 > F1 accordingly. Thus, it was concluded that F4 showed the highest bioadhesive strength as in column 6 of Table 2.

**Table 2:** % Yield, drug content, extrudability, spreadability, and bioadhesive strength of bioadhesive vaginal gel

Formulation code	% Yield*	Drug Content (mg/100 g of gel)*	Extrudability (g/cm^2^)*	Spreadability (g.cm/sec)*	Bioadhesive strength (g)*
F1	99.85 ± 1.05	96.24 ± 0.09	94 ± 1.02	135.43 ± 0.31	43.41 ± 0.05
F2	99.37 ± 1.26	98.81 ± 0.65	97 ± 2.93	127.36 ± 0.24	45.33 ± 0.03
F3	99.90 ± 0.05	98.84 ± 0.01	89 ± 0.28	158.12 ± 0.24	55.07 ± 0.02
F4	98.97 ± 3.36	100.01 ± 0.19	84 ± 1.78	119.29 ± 0.02	60.12 ± 0.01

* All values are expressed in mean ± standard deviation (*n* = 3)

### *In vitro* drug release and drug release kinetics of gel formulations

**Figure 1:**
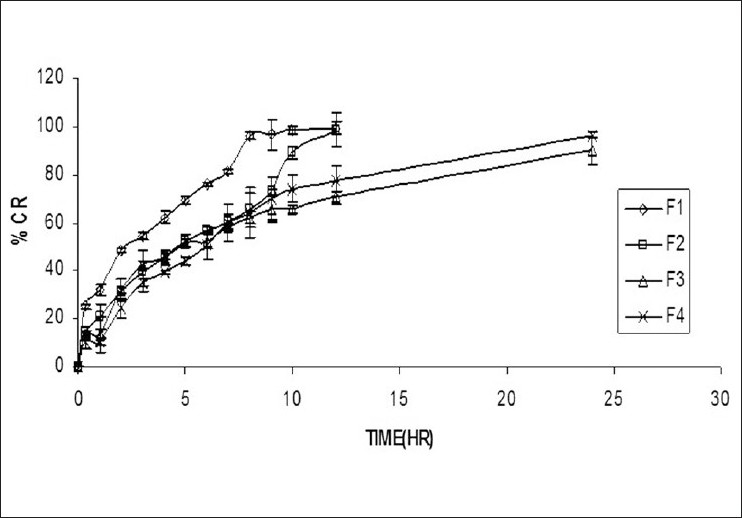
Drug diffusion profiles of bioadhesive vaginal gel (F1–F4)

Figure 1 and Table 3 compares the *in vitro* drug release profiles of all the four formulations. It can be seen that the drug release throughout the study period was more or less steady. Formulations F1 and F2, where Carbopol P940 and HPMC combination (1:1 and 1:2) were used, showed the *t*_90_ (time taken for 90% of drug released) 8 and 10 h, respectively. The data obtained suggest that the concentration of the polymer used is inadequate to control the release of AZT. Separately, in the next stage, attempt was made to control the release using vaginal gel into the vagina. It is of profound interest that the release profile of formulations F3 and F4 with Carbopol P940 and HPMC combination (1:3 and 1:4) showed a drug release throughout the study period as desired. *t*_90_ was found to be 24 and 20 h for F3 and F4, and it was significantly different (P < 0.05) among the formulations. All the formulations were also analyzed for the drug release kinetics. Although drug release from all the bioadhesive vaginal gel formulations (F1–F4) was observed up to 24 h, the drug release mechanism of all Fs was found to be predominately influenced by the different bioadhesive polymers added. The mechanism of drug release from hydrophilic polymeric matrices involves solvent penetration, hydration and swelling of the polymer, diffusion of the dissolved drug in the matrix and erosion of the gel layer. On the basis of the Diffusion control studies Table 3 and Figure 1, it was observed that the F1 (*n* = 0.447) and F3 (*n* = 0.514) underwent case I Fickian diffusion control, during the dissolution study. In case of Fickian release mechanism, the rate of drug release is much lesser with polymer relaxation (swelling/erosion). Therefore, the drug release was chiefly dependent on the diffusion through the matrix. Also, it was observed that the bioadhesive vaginal gels F2 (*n* = 0.564) and F4 (*n*=0.652) from column 10 of Table 3, underwent non-Fickian (anomalous) diffusion control during the drug release study, which indicated that polymer relaxation had a significant role in the drug release mechanism. In the non-Fickian (anomalous) case II release, the rate of drug release is due to the combined effect of drug diffusion and polymer relaxation. Nature of release of the drug from the vaginal gels was inferred based on the correlation coefficients obtained from the plots of the kinetic models.

On the basis of excellent bioadhesive strength (55.07 ± 0.02 g), high drug content (98.84 ±0.01% mg/100g of gel, soft in nature, extrudability (89 g ± 0.28 g/cm^2^), spreadability (158.12 ° 0.24 g cm/s) and good release profile (released only 90.18 ± 0.03% up to 24 h in a constant manner) bioadhesive vaginal gel formulation F3 was selected for further studies.

**Table 3:** Drug release and drug release kinetics of bioadhesive Vaginal Gel

Formulations	Cumulative % Drug release, %CR*	Zero-order release, K_o_		First-order release		Higuchi square root equation		Korsmeyer and Peppas equation	
		*K*_0_	*r*_2_	*K*_1_	*r*_2_	*K*_h_	*r*_2_	*r*_2_	*n*
F1	99.42 ± 0.52 (12 h)	7.74	0.89	0.18	0.91	30.25	0.98	0.98	0.447
F2	98.67 ± 0.05 (12 h)	7.32	0.96	0.12	0.67	27.25	0.96	0.99	0.564
F3	90.18 ± 0.03 (24 h)	3.05	0.66	0.04	0.97	17.64	0.94	0.99	0.514
F4	96.45 ± 0.02 (24 h)	4.09	0.82	0.08	0.79	22.16	0.97	0.99	0.652

* All values are expressed in mean ± standard deviation (*n* = 3)

### *In vivo* studies of F3

**Figure 2:**
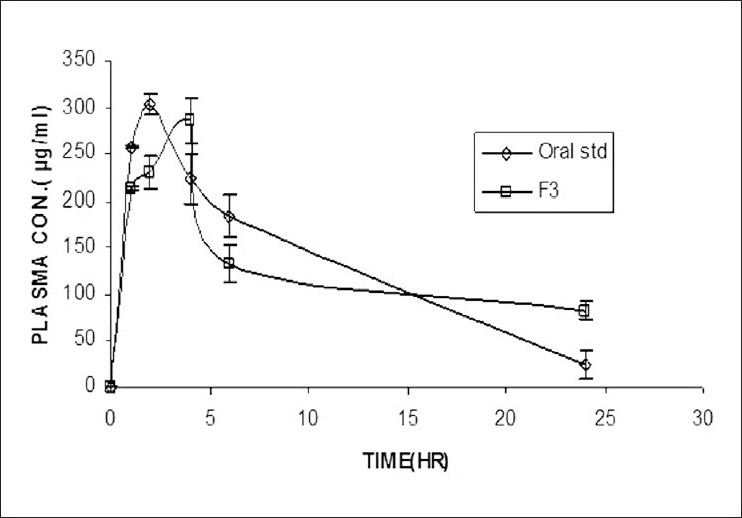
Plasma drug concentration profiles of bioadhesive vaginal gel with oral standard

F3 formulation containing the ratio (1:3) and pure drug solution (1 mg/mL) after oral administration were used to plot percent plasma concentrations versus time curve [Figure 2] for Zidovudine. Pharmacokinetic parameters for both are presented in Table 4. F3 formulation of AZT showed (*C*_max_) 17.659 μg mL ^-1^ in blood as compared to pure drug solution 18.494 μg mL ^-1^ and bioavailability (*F*) 123.89% as compared with pure drug solution (considered 100%). *T*_max_ of F3 was 4 h, and pure drug solution was 2 h. AUC is an important parameter for evaluating bioavailability of drug from dosage form as it represents the total integrated area under the blood concentration time profile and represents the total amount of drug reaching. The F3 formulation and pure drug solution wear not a statistically significantly different (*P* < 0.05) indicated that drug amount between the formulations did not vary.

**Table 4:** Pharmacokinetic parameter comparison with oral standard of bioadhesive vaginal gel

Parameter	Standard dose (oral)	Bioadhesive vaginal gel (F3)
C_max_	18.494 μg mL-1	17.659 μg mL-1
T_max_	2 h	4 h
$\int_{0}^{\infty }$*AUC*(μg h / mL)^4^	182.02 μg h2 mL^-1^	225.507 μg h2 mL^-1^
$\int_{0}^{\infty }$*AUMC* (μg h^2^ / mL)	848.23 μg h2 mL^-1^	1831.377 μg h2 mL^-1^
MRT	4.66h	8.21 h
T1/2	1 h	1 h
KE	0.693h	0.693 h
Vd	0.680 lt/kg	0.070 lt/kg
Cl	0.047 lt/kg/h	0.48 lt/kg/h
Bioavailability (*F*)	100 (consider)	123.89%
Dose administered	2 mg	2 mg

## CONCLUSION

F3 formulation containing carbopol–HPMC (1:3) was selected and evaluated for this study. *In vitro* drug release study of F3 showed t_90_ in 24h drug released following case I Fickian (n≤0.5) transport mechanism. In case of *in vivo* drug release it was found that (T_max_) 4h and (Cmax) 17.659 μg ml-1, bioavailability (F) 123.89 % comparison with oral standard drug solution. It also shows good extrudability, spreadability, and good bioadhesive strength. Further research in this area will surely expected to yield significant outcome with improved vaginal drug delivery system for the treatment of AIDS and its prevention.
